# Effective Photocatalytic Activity of Sulfate-Modified BiVO_4_ for the Decomposition of Methylene Blue Under LED Visible Light

**DOI:** 10.3390/ma12172681

**Published:** 2019-08-22

**Authors:** Vinh Huu Nguyen, Quynh Thi Phuong Bui, Dai-Viet N. Vo, Kwon Taek Lim, Long Giang Bach, Sy Trung Do, Tuyen Van Nguyen, Van-Dat Doan, Thanh-Danh Nguyen, Trinh Duy Nguyen

**Affiliations:** 1 Center of Excellence for Green Energy and Environmental Nanomaterials (CE@GrEEN), Nguyen Tat Thanh University, Ho Chi Minh 755414, Vietnam; 2 NTT Hi-Tech Institute, Nguyen Tat Thanh University, Ho Chi Minh 755414, Vietnam; 3 Faculty of Chemical Technology, Ho Chi Minh City University of Food Industry, Ho Chi Minh 705800, Vietnam; 4 Department of Display Engineering, Pukyong National University, Busan 608-737, Korea; 5 Institute of Chemistry, Vietnam Academy of Science and Techology, Hanoi 10072, Vietnam; 6 Faculty of Chemical Engineering, Industrial University of Ho Chi Minh city, Ho Chi Minh 700000, Vietnam; 7 Institute of Research and Development, Duy Tan University, Da Nang City 550000, Vietnam; 8 Institute of Chemical Technology, Vietnam Academy of Science and Technology, Ho Chi Minh 700000, Vietnam

**Keywords:** sulfate-modified BiVO_4_, methylene blue, LED visible light, photodecomposition

## Abstract

In this study, we investigated sulfate-modified BiVO_4_ with the high photocatalytic activity synthesized by a sol-gel method in the presence of thiourea, followed by the annealing process at different temperatures. Its structure was characterized by thermal gravimetric analysis (TGA), powder X-ray diffraction (XRD), Raman spectroscopy, scanning electron microscopy/energy-dispersive X-ray spectroscopy (SEM/EDS), X-ray photoelectron spectroscopy (XPS), and ultraviolet-visible diffuse reflectance spectroscopy (UV-Vis DRS). The BiVO_4_ synthesized in the presence of thiourea and calcined at 600 °C (T-BVO-600) exhibited the highest photocatalytic degradation efficiency of methylene blue (MB) in water; 98.53% MB removal was achieved within 240 min. The reaction mechanisms that affect MB photocatalytic degradation on the T-BVO-600 were investigated via an indirect chemical probe method, using chemical agents to capture the active species produced during the early stages of photocatalysis, including 1,4-benzoquinone (scavenger for O_2_^−^), ethylenediaminetetraacetic acid disodium salt (scavenger for h^+^), and *tert*-butanol (scavenger for HO^•^). The results show that holes (h^+^) and hydroxyl radicals (HO^•^) are the dominant species of MB decomposition. Photoluminescence (PL) measurement results of terephthalic acid solutions in the presence of BiVO_4_ samples and BiVO_4_ powders confirm the involvement of hydroxyl radicals and the separation efficiency of electron-hole pairs in MB photocatalytic degradation. Besides, the T-BVO-600 exhibits good recyclability for MB removal, achieving a removal rate of above 83% after five cycles. The T-BVO-600 has the features of high efficiency and good recyclability for MB photocatalytic degradation. These results provide new insight into the purpose of improving the photocatalytic activity of BiVO_4_ catalyst.

## 1. Introduction

Bismuth vanadate (BiVO_4_) has recently been extensively studied by researchers around the world and has been used as a new catalyst in the photocatalytic field because of the economic advantage of synthetic materials, low toxicity, excellent chemical stability, and narrow bandgap (about 2.4 eV for monoclinic scheelite BiVO_4_) [1]. Researchers have discovered that BiVO_4_ offers outstanding photocatalytic performance in water splitting and oxidation of toxic organic compounds [2,3,4,5,6]. The photoinduced charge carrier formation was highly efficient due to low bandgap energy properties. However, recombination of excess electrons and holes are extensive due to its poorly charged transfer characteristics and weakly adsorbed surface, limiting the photocatalytic activity of BiVO_4_ [7]. In order to improve the separation efficiency of the photogenerated electron-hole pairs to the catalytic surface for high-photodynamic catalysis, the researchers proposed several measures, such as (1) control of crystal structure, crystal form, and crystal surface [8,9], (2) formation of p-n bonds and the establishment of an internal electromagnetic interaction region extending from n-type semiconductor (BiVO_4_) to p-type semiconductor materials [10], and (3) formation of the monoclinic-tetragonal structure of BiVO_4_ [11]. Such approaches mainly involve improving the photocatalytic activity of BiVO_4_, which is enhanced through either material synthesis with crystal form control or doping with nonmetal elements which have been proven to be efficient and promising research directions recently.

According to previously published studies, the concentration of the reactants and the solution medium (such as the pH, the effect of anions) had significant implications for the crystalline form of BiVO_4_ in solution. For example, small changes in the affecting factors (such as pH, temperature, and reactants) will alter the growth of BiVO_4_ crystals. As a result, crystalline forms such as nanoplates [12], micro bar [13], elliptic structure [14], and various crystal forms are formed [15]. Therefore, the addition of a surfactant to the reaction solution to control the crystal growth process could facilitate the designing of the ideal catalyst. For this purpose, recently, researchers have been using urea for the synthesis of BiVO_4_. Urea can control the precipitation of cation by slowly forming hydroxide ions in solution through hydrolysis [9,16]. Thus, the slow hydrolysis of urea leads to a gradual increase in the pH of the reaction solution and provides a special solution for controlling the crystal growth process. Also, the doping of nonmetal elements, such as S [17], C [18], N [19], and P [20], into the structure of BiVO_4_ photocatalyst also enhances the photocatalytic efficiency of this material. Similar to urea, thiourea (CS(NH_2_)_2_) also hydrolyzes to form NH_3_, which participates in the pH adjustment of the reaction solution, thus contributing to the process of controlling the crystal growth of the material. Also, previous studies have used thiourea as the S source to modify BiVO_4_ to enhance the photocatalytic activity of the material [4,17,21]. The previous studies have used thiourea or Na_2_S as the S source to modify BiVO_4_ to enhance the photocatalytic activity of the material. However, S-doped BiVO_4_ was synthesized by hydrothermal method, and surfactants were introduced in the synthesis process. In some of these studies, these surfactants may have a bad effect on the environment. Therefore, easier methods to synthesize S-doped BiVO_4_ are necessary.

Hence, we report on the synthesis of sulfate-modified BiVO_4_ by a sol-gel method using thiourea as the reducing agent to control simultaneous crystal morphology as well as the S source for material modification. At the same time, we investigated the effect of heating temperature on the structure of BiVO_4_ as well as on the photocatalytic activity of the material in the decomposition of organic compounds using visible light.

## 2. Experimental

### 2.1. Materials

Ammonium metavanadate (NH_4_VO_3_, ≥98%), bismuth(III) nitrate pentahydrate (Bi(NO_3_)_3_·5H_2_O, ≥98.0%), *tert*-butanol (TBA, (CH_3_)_3_COH, ≥99.5%), and 1,4-benzoquinone (BQ, C_6_H_4_O_2_) were purchased from Sigma-Aldrich. Thiourea (CH_4_N_2_S, 99.8%) was purchased from Prolabo (France). Nitric acid (HNO_3_, 65–68%), ethanol (CH_3_CH_2_OH, 99.7%), ethylenediaminetetraacetic acid disodium salt (EDTA-2Na), and methylene blue (MB, 99%) were obtained from Xilong Chemical Co., Ltd. (Shantou, China).

### 2.2. Fabrication of Sulfate-Modified Bismuth Vanadate (BiVO_4_)

We synthesized the sulfate-modified BiVO_4_ using the sol-gel combustion method with a coupling of the sol-gel process and the annealing process. Firstly, 20 mmol of Bi(NO_3_)_3_·5H_2_O was dissolved in 200 mL of HNO_3_ (2M) and stirred for about 30 min to form a clear solution (solution A). At the same time, 20 mmol of NH_4_VO_3_ was dissolved in 200 mL of water and stirred for about 180 min at 70 °C to form a uniform transparent yellow solution (solution B). After adding solution B to solution A drop by drop, a dark yellow solution was obtained. Then, 62.5 mmol of thiourea was added to the mixture. The obtained mixture was vigorously stirred for 30 min before being heated at 85 °C to evaporate the water under continuous stirring overnight. Finally, the obtained powder was finely ground and calcined at different temperatures (400–700 °C) for 3 h with a heating rate of 5 °C/min in the air. The obtained samples were denoted as T-BVO-400, T-BVO-500, T-BVO-600, and T-BVO-700, corresponding to the annealing temperature of 400, 500, 600, and 700 °C, respectively. For comparison, BiVO_4_ was synthesized with the absence of thiourea and calcined at 600 °C (denoted as BVO-600).

### 2.3. Characterization

Thermal gravimetric analysis (TGA) was conducted on a TGA Q500 V20.10 Build 36 under air condition with a heating rate of 5 °C/min from room temperature to 800 °C. X-ray diffraction (XRD) patterns were recorded in a D8 Advance Bruker powder diffractometer (Bruker, Billerica, MA, USA) with a Cu Kα excitation source at a scan rate of 0.030°/s in the 2-theta range of 5–80°. The surface morphologies and particle size of BiVO_4_ samples were observed by scanning electron microscope (SEM, JEOL JSM 7401F, Peabody, MA, USA). X-ray photoelectron spectroscopy (XPS) was recorded on Thermo VG Multilab 2000 (Thermo VG Scientific, Waltham, MA, USA). Fourier transform infrared (FT-IR) spectra were recorded on an EQUINOX 55 spectrometer (Bruker, Billerica, MA, USA). Raman spectroscopy was carried out on the HORIBA Jobin Yvon spectrometer (Horiba Scientific, Kyoto, Japan) with a laser beam of 633 nm in the wavenumber of 100–1000 cm^−1^. The optical absorption characteristics of the photocatalysts were determined by ultraviolet-visible (UV-Vis) diffuse reflectance spectroscopy (UV-Vis DRS, Shimazu UV-2450, Kyoto, Japan) in the range of 300–900 cm^−1^. Photoluminescence (PL) measurements were recorded using an F-4500 Spectro-fluorometer (Hitachi, Chiyoda, Japan).

### 2.4. Photocatalytic Activity Test

The photocatalytic activities of the BiVO_4_ samples were evaluated by the photodegradation of methylene blue (MB) in a 250 mL double-layer interbed glass beaker photocatalytic reactor under visible light irradiation by six daylight Cree^®^ Xlamp^®^ XM-L2 LEDs (Cree, Inc., Durham, NC, USA) with max power of 10 W and max light output of 1052 lumen). In each run, a mixture consisting of MB aqueous solution (15 ppm, 100 mL) and the given catalyst (100 mg) was magnetically stirred in the dark for 1 h to reach the adsorption-desorption equilibrium of the dye on the catalyst surface. After this time, the LEDs light source was switched on. Five mL of the suspension was withdrawn at the same intervals and immediately centrifuged to separate photocatalyst particles at 7000 rpm for 15 min. The MB concentration was monitored by measuring the absorption intensity at its maximum absorbance wavelength of λ = 664 nm using a UV-visible spectrophotometer (Model Evolution 60S, Thermo Fisher Scientific, Massachusetts, MA, USA) in a 1 cm path length spectrometric quartz cell.

### 2.5. Active Species Trapping Experiments

The active species generated during the early stages of photocatalytic processes such as O_2_^−^, h^+^, and HO^•^ are responsible for MB degradation and are determined by an indirect chemical probe method. Chemical agents, namely BQ, EDTA-2Na, and TBA were agents that capture O_2_^−^, h^+^, and HO^•^, respectively. The experimental procedure was similar to the above photocatalytic experiments except that the chemical agents are added before the beginning of the photocatalytic experiment. The concentration of agents added was 0.3 M except for BQ. The concentration of BQ was 1.0 × 10^−3^ M because higher concentrations of BQ might hinder the determination of MB concentration by UV-Vis absorption spectra.

### 2.6. Analysis of Hydroxyl Radical (HO^•^)

The generation of hydroxyl radicals (HO^•^) in the LED/sulfate-modified BiVO_4_ system was detected by the PL technique using terephthalic acid (TA) as the sensor molecule. The formation of 2-hydroxyterephthalic acid (HTA) from the reaction of TA with HO^•^ radicals exhibits strong photoluminescence. The process was similar to the above photocatalytic activity test with the replacement of MB solution by TA solution containing TA 0.5 mM and NaOH 2 mM. A fluorescence spectrophotometer analyzed the clear solution at an excitation wavelength of 315 nm after 240 min of irradiation.

## 3. Results and Discussion

### 3.1. Characterization of Sulfate-Modified BiVO_4_ and Pure BiVO_4_

**Figure 1:**
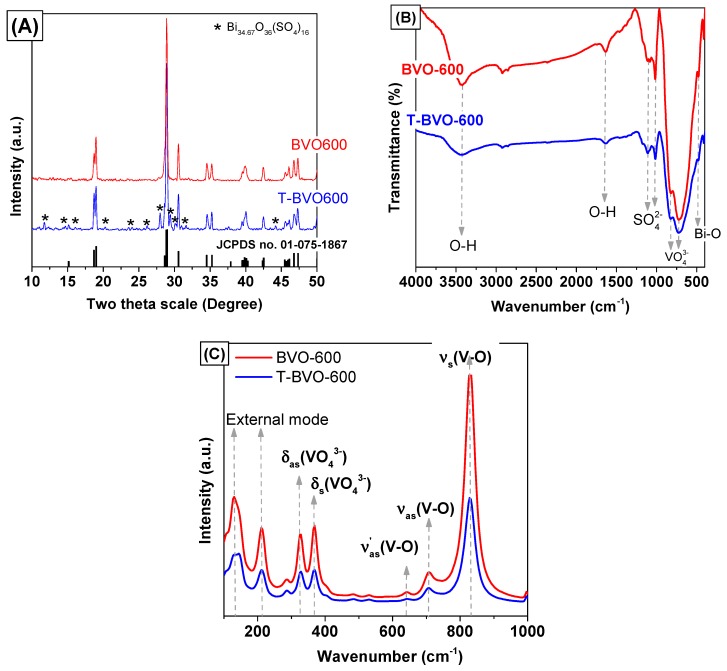
The X-ray diffraction (XRD) patterns (**A**), Fourier transform infrared (FT-IR) spectra (**B**), and Raman spectra (**C**) of T-BVO-600 and BVO-600.

The crystal structure of sulfate-modified BiVO_4_ and pure BiVO_4_ was confirmed by X-ray diffraction. Figure 1A displays the XRD pattern of the BiVO_4_ samples that were synthesized with/without the presence of thiourea and calcined at 600 °C (T-BVO-600 and BVO-600 samples). For the BVO-600 sample, the diffraction peaks on the XRD pattern aligned with the monoclinic scheelite phase of BiVO_4_ (m-s BiVO_4_, JCPDS no. 01-075-1867) with weakly diffracted peaks at 2θ = 15.5°, strongly diffracted peaks at 2θ = 28.9°, and the splitting of peaks at 2θ = 18.5°, 35°, and 47°. However, with the presence of thiourea, the XRD pattern of the T-BVO-600 sample not only shows the characteristic diffraction peaks in the monoclinic scheelite structure, but also exhibits weak diffraction peak characteristics for bismuth oxide sulfate (Bi_34.67_O_36_(SO_4_)_16_, JCPDS no. 00-041-0689), indicating that the presence of thiourea did not alter the phase structure of m-s BiVO_4_ crystal. In addition, there was no observable change in the position of the (121) and (040) planes of both T-BVO-600 and BVO-600 samples, implying that S could not be doped into the lattice of BiVO_4_ crystal. The negligible differences between lattice parameters of T-BVO-600 and BVO-600, which are shown in Table 1, also confirm that no significant change in the crystal phase of BiVO_4_ occurred. These analyses indicated the possible loading of S to the BiVO_4_ surface as sulfate instead of doping into the lattice of BiVO_4_.

To investigate the presence of functional groups and bonds in the material structure, the material was analyzed by FT-IR, as shown in Figure 1B. In general, both T-BVO-600 and BVO-600 samples have characteristic vibration peaks for m-s BiVO_4_, which is consistent with the FT-IR results for the BiVO_4_ material in the previous study. These peaks are the stretching vibration (δ) and bending vibration mode (υ) respectively, of the O–H bond of the water molecules adsorbed onto the surface of the material [22], the bending vibration of the Bi–O bond [23], the stretching vibration of the ${\mathrm{VO}}_{4}^{3-}$ group [22,23], and the asymmetric stretching vibration of V=O [24]. Also, the presence of the ${\mathrm{SO}}_{4}^{2-}$ group on the T-BVO-600 sample was detected through FT-IR spectra at wavenumbers of 1110 and 1016 cm^−1^, corresponding to the asymmetric and symmetric stretching vibrations of S=O bonds [25].

Figure 1C displays the Raman spectra of sulfate-modified BiVO_4_ and pure BiVO_4_. As shown in Figure 1C, both samples have characteristic vibration peaks for m-s BiVO_4_, consisting of (i) the stretching vibration (δ_s_) and asymmetric vibration mode (δ_as_) of V–O at 829 and 709 cm^−1^ respectively, (ii) the symmetrical bending vibrations (υ_s_) and asymmetric bending vibrations (υ_as_) of the ${\mathrm{VO}}_{4}^{3-}$ group at 368 and 327 cm^−1^ respectively, and (iii) the external modes (rotation/translation) in BiVO_4_ at 212 and 129 cm^−1^ [26]. The V–O bond length of sulfate-modified BiVO_4_ and pure BiVO_4_ samples can be calculated via the empirical expression [27,28]:

(1) $$\nu =21349\cdot e^{-19176\cdot R}$$

where, ν is the stretching vibration frequency of V–O (cm^−1^) and R is the V–O bond length (Å). The R values of T-BVO-600 and BVO-600 samples are 1.694 and 1.692 Å, respectively. This result further confirmed the monoclinic scheelite phase of BiVO_4_.

**Figure 2:**
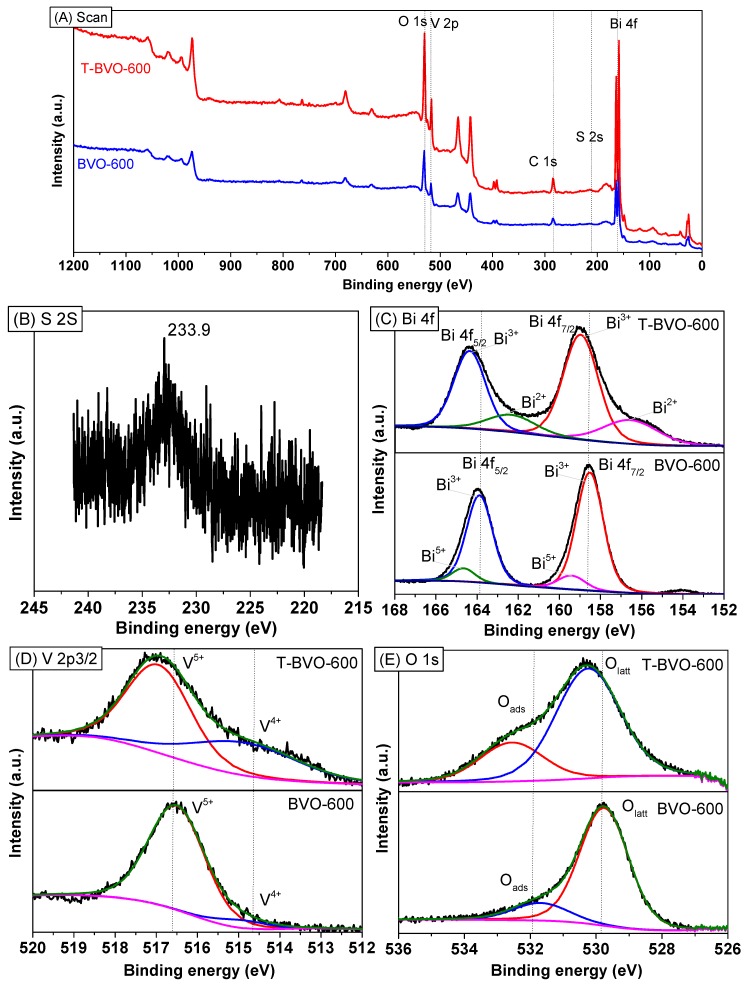
Full scan (**A**), S 2s (**B**), Bi 4f (**C**), V 2p3/2 (**D**), and O 1s (**E**) X-ray photoelectron spectroscopy (XPS) spectra of T-BVO-600 and BVO-600.

To provide more structure information, the chemical states of T-BVO-600 and BVO-600 samples were determined by XPS. As shown in Figure 2A, the chemical composition of the two samples mainly consists of Bi, V, C, and O elements. The presence of S element in the T-BVO-600 sample indicated that S is deposited on the BiVO_4_ surface. The high-resolution XPS spectrum of the T-BVO-600 showed a broad peak of S 2 s at 233.9 eV (Figure 2B), assigned to S^+6^ in the ${\mathrm{SO}}_{4}^{2-}$ group [29,30]. Figure 2C shows the Bi 4f spectrum containing two strongly symmetric peaks Bi 4f_7/2_ and Bi 4f_5/2_. They are 158.97 eV and 164.36 eV for the T-BVO-600 sample, and 158.52 eV and 163.88 eV for the BVO-600 sample respectively, which are characteristics of Bi^3+^ [31,32]. The shoulder of the Bi 4f_7/2_ (159.41 eV) and Bi 4f_5/2_ (164.66 eV) spectra for the BVO-600 sample could be ascribed to the presence of Bi^5+^ oxidation state in BiVO_4_ [33,34]. The shoulder of the Bi 4f_7/2_ (156.55 eV) and Bi 4f_5/2_ (162.34 eV) spectra for the T-BVO-600 sample could be ascribed to the presence of Bi^2+^ oxidation state in BiO [35,36]. The asymmetric V 2p_3/2_ signals can be decomposed into two subpeaks at 514.85 eV and 516.95 eV for T-BVO-600, and 514.9 and 516.51 eV for BVO-600 (Figure 2C), which is assigned for V^4+^ and V^5+^ species, respectively [31]. The surface molar of V^4+^/V^5+^ for T-BVO-600 (0.502) was higher than that of the BVO-600 (0.027) sample, which confirmed that the T-BVO-600 sample was oxygen-deficient [19]. The BE positions of O 1s at 530.10 eV in the BVO-600 sample and 530.9 eV in the T-BVO-600 sample (Figure 2D) are assigned to O^2−^ [17]. In the case of BVO-600, this peak can be decomposed into two subpeaks at 529.76 eV and 531.69 eV (Figure 2D), which are assigned to the lattice oxygen (O_latt_) in crystalline BiVO_4_ and the adsorbed oxygen (O_ads_) on the BiVO_4_ surface, respectively. Similarly, the O 1s peak of T-BVO-600 can also be decomposed into two subpeaks at 530.25 eV and 532.57 eV (Figure 2D). However, the slight shifting of the peak indexed to the adsorbed oxygen (O_ads_) toward a higher BE for the T-BVO-600 (Figure 2D) could be due to the presence of sulfate on the BiVO_4_ surface. The surface molar of O_ads_/O_latt_ for T-BVO-600 (0.354) was lower than that of the BVO-600 sample (0.436). The low number of O_ads_ species play an important role in the photocatalytic performance of T-BVO-600 (to be seen in investigating the mechanisms of dye degradation section). The XPS results, together with XRD and FT-IR, confirm the presence of bismuth oxide sulfate (Bi_34.67_O_36_(SO_4_)_16_) in the T-BVO-600 structure.

**Figure 3:**
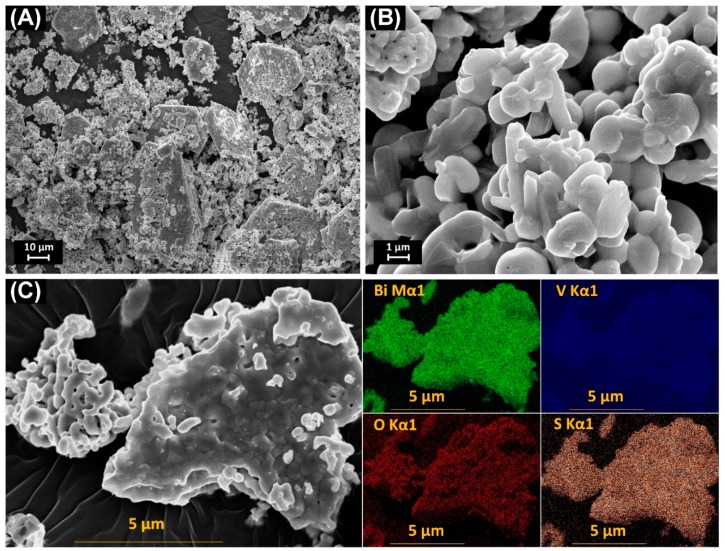
Scanning electron microscopy (SEM) images the BiVO_4_ samples: T-BVO-600 (**A**) and BVO-600 (**B**) and field emission scanning electron microscopes and energy-dispersive X-ray spectrometer (FESEM/EDS) images of the T-BVO-600 sample with maps of Bi Ma1, V Ka1, OKa1, and S Ka1 (**C**).

Crystal morphology, particle size, and particle distribution of the material were observed through SEM images. The SEM images of the samples were synthesized under different conditions, as shown in Figure 3A,B. The obtained shape and crystal size of the materials were very different when synthesizing under different conditions. For the BiVO_4_ sample which was synthesized without thiourea and calcined at 600 °C, the crystal morphology has a granular shape and is approximately 1 μm in size. For the BiVO_4_ synthesized using thiourea, the forming material has a granular crystalline form with insignificant granular boundaries, and particles are deposited into large plates with openings formed between the particles. In addition, the presence of S element in the T-BVO-600 sample was also confirmed by FE-SEM/EDS images (Figure 3C). These results, along with the XPS result (Table 2), revealed that sulfate was successfully deposited into the BiVO_4_ surface.

**Figure 4:**
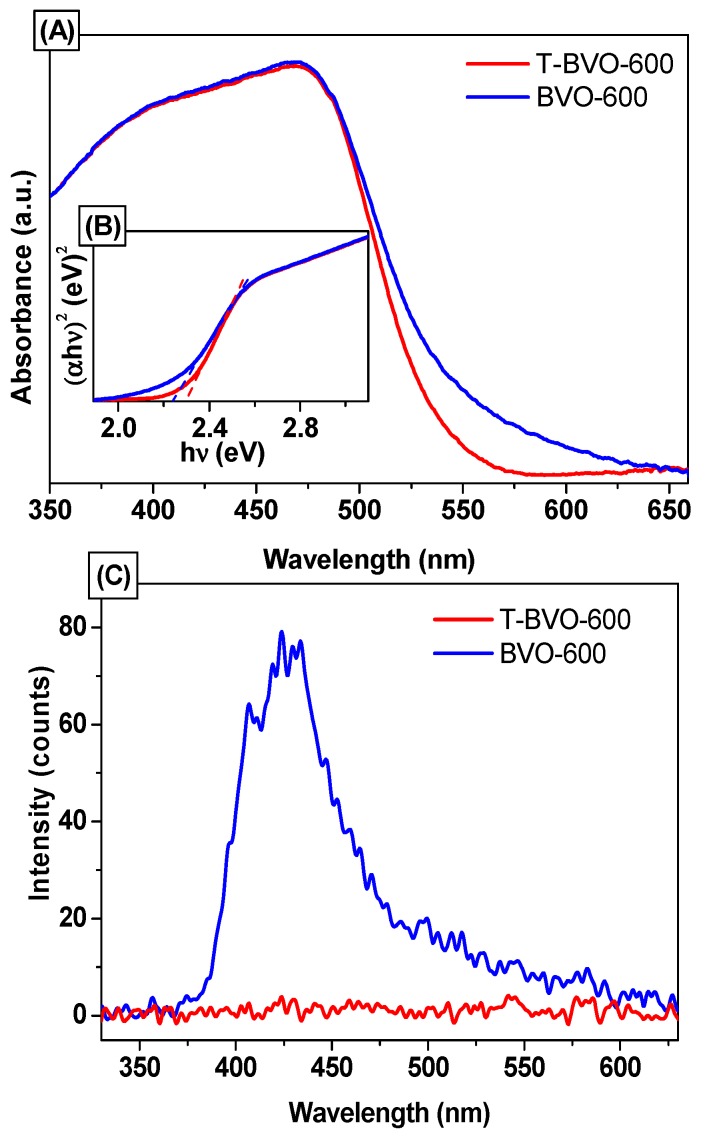
Ultraviolet-visible diffuse reflectance spectroscopy (UV-Vis DRS) spectra (**A**), (ahν)^2^–hν curves (**B**), and photoluminescence (PL) spectra of T-BVO-600 and BVO-600 (**C**).

The light absorption properties of photocatalytic materials were analyzed using the UV-Vis DRS technique. The results are shown in Figure 4A. All BiVO_4_ samples showed narrow absorption in the visible light region, which can facilitate the enhancement of photocatalytic property under visible light irradiation. The bandgap energy of BiVO_4_ samples can be estimated from the (ahν)^2^–hν curves (Figure 4B). The E_g_ value of the BiVO_4_ samples was determined to be 2.29 eV for T-BVO-600 and 2.23 eV for BVO-600.

The photoluminescence spectra of the T-BVO-600 and BVO-600 samples were also used to study the separation efficiency of electron-hole pairs generated by irradiation. The higher intensity of the PL spectrum of the sample indicates that the electron-hole pair recombination takes place more rapidly, thus reducing photocatalytic activity. The PL spectrum of the BiVO_4_ samples upon excitation at 325 nm is shown in Figure 4C. It can be seen that BVO-600 generates broad emission peaks in the wavelength range of 360–625 nm with a maximum emissivity of 430 nm corresponding to the transfer of charges from Bi to V centers [37]. Whereas the synthetic BiVO_4_ samples using thiourea do not seem to emit peaks in this wavelength range, indicating that the recombination of electrons and holes in these samples is very low.

### 3.2. Effect of the Annealing Temperature

**Figure 5:**
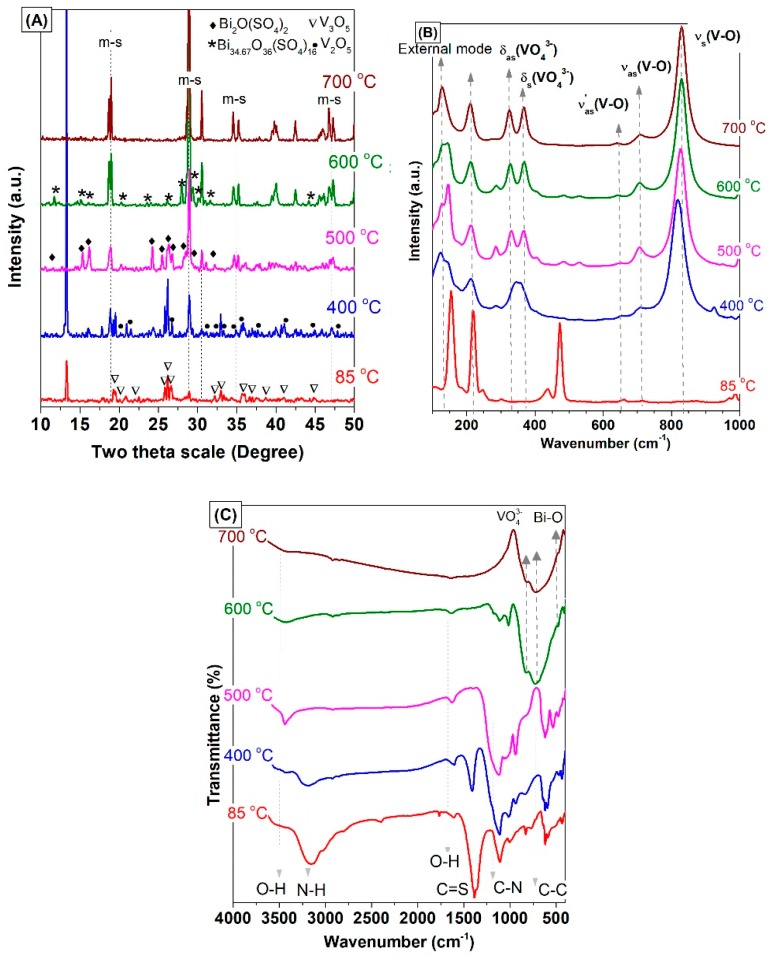
XRD pattern (**A**), Raman spectra (**B**), and FT-IR spectra (**C**) of the BiVO4 amorphous (85 °C) and sulfate-modified BiVO_4_ annealed at 400, 500, 600, and 700 °C.

To investigate the effect of the annealing temperature on the phase structure of BiVO_4_ crystals, the amorphous BiVO_4_, which was obtained after heating at 85 °C under continuous stirring overnight (T-BVO-85), was calcined at a different temperature. XRD patterns of the amorphous BiVO_4_ and BiVO_4_ samples annealed from 400 °C to 700 °C are shown in Figure 5A. As shown in Figure 5A, the amorphous BiVO_4_ exhibited a low crystal with the main phase of vanadium oxide (V_3_O_5_, JCPDS No. 01-071-0039). When T-BVO-85 was annealed at 400 °C, the XRD pattern showed the coexistence of tetragonal scheelite phase of BiVO_4_ (t-s BiVO_4_, JCPDS No. 14-0133) and V_2_O_5_ (JCPDS No. 00-041-1426). Besides, we also observed that the peak at 2θ = 13.26° for T-BVO-85 and T-BVO-400 samples were related to the typical in-planar peak of graphitic carbon nitride (g-C_3_N_4_) polymers [38,39] which were formed during heating at 85 °C to evaporate the water and the annealing at 400 °C. When further increasing the annealing temperature to 500 °C (T-BVO-500), the XRD pattern of this sample demonstrated a pattern similar to that of m-s BiVO_4_ with the coexistence of Bi_2_O(SO_4_)_2_ (JCPDS No. 01-078-2087). The peak at 13.26° disappeared, which indicated that the organic compounds were completely burned. The T-BVO-600 sample exhibited the coexistence of m-s BiVO_4_ and Bi_34.67_O_36_(SO_4_)_16_, which was analyzed above. When further increasing the annealing temperature to 700 °C, pure m-s BiVO_4_ was formed. The XRD results indicate that the different annealing temperatures would result in BiVO_4_ materials with different composition and phase.

The FT-IR spectra of the amorphous BiVO_4_ and BiVO_4_ samples annealed from 400 °C to 700 °C were shown in Figure 5B. For the amorphous BiVO_4_ and BiVO_4_ samples annealed at 400 °C and 500 °C, absorption bands are located at 620, 730, 806, 940, 1110, 1385, 1406 (and 1598), and 3168 cm^−1^ assigned to the C–C (aromatic), N–H (bending), tri-s-triazine, C–H, C–C, C–H (aromatic), C–N (aromatic), and N–H vibrations respectively, which confirms the formation of g-C_3_N_4_ [40,41,42,43]. The formation of g-C_3_N_4_ compounds obtained by polycondensation of thiourea at different temperatures is in agreement with previous reports [38,44,45]. The BiVO_4_ samples annealed at 600 °C and 700 °C have characteristic vibration peaks for m-s BiVO_4_ and no peaks that could be indexed to the presence of organic compounds. 

The Raman results (Figure 5C) show that the BiVO_4_ samples annealed at 500 °C, 600 °C, and 700 °C have characteristic vibration mode in the m-s BiVO_4_ while T-BVO-400 has characteristic vibration mode in the t-s BiVO_4_ [26]. In addition, the R values of the T-BVO-500 and T-BVO-600 samples shown in Table 1 matched with the V–O bond length of m-s BiVO_4_, while the R values of T-BVO-400 matched with the V–O bond length of m-s BiVO_4_ [46]. This result further confirmed the significant effect of annealing temperature on the structure phase of BiVO_4_, which is in agreement with XRD and IR results. 

**Figure 6:**
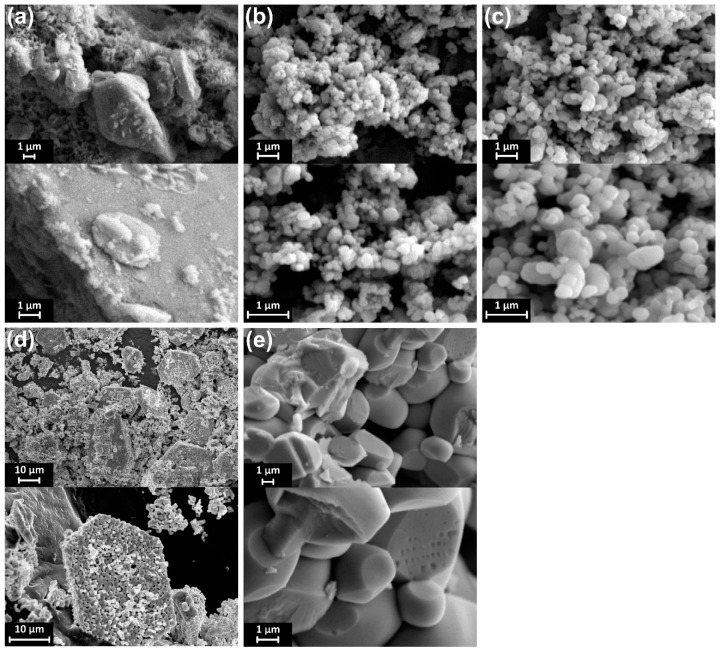
SEM images of the BiVO4 amorphous (**a**) and sulfate-modified BiVO_4_ annealed at 400 °C (**b**), 500 °C (**c**), 600 °C (**d**), and 700 °C (**e**).

The SEM images of the samples synthesized under different conditions are shown in Figure 6. The obtained shape and crystal size of the materials were very different when synthesized under different conditions. For the BiVO_4_ synthesized using thiourea, the non-calcined sample has no definite shape. After being heated at 400 °C and 500 °C, the crystals with granular shape are formed and sized less than 1 μm. The particle size becomes more significant with increasing calcining temperature (Figure 6) due to the growth of BiVO_4_ crystals during high calcining temperature. When the calcining temperature increases to 600 °C, the forming material has a granular crystalline form with insignificant granular boundaries and particles are deposited into large plates with openings formed between the particles. At 700 °C, the material forming the crystal structure is mainly irregular particles, about 2 μm in size. Obviously, heat treatment exerts a significant influence on the morphology and crystallinity of BiVO_4_ when the BiVO_4_ was synthesized in the presence of thiourea. 

**Figure 7:**
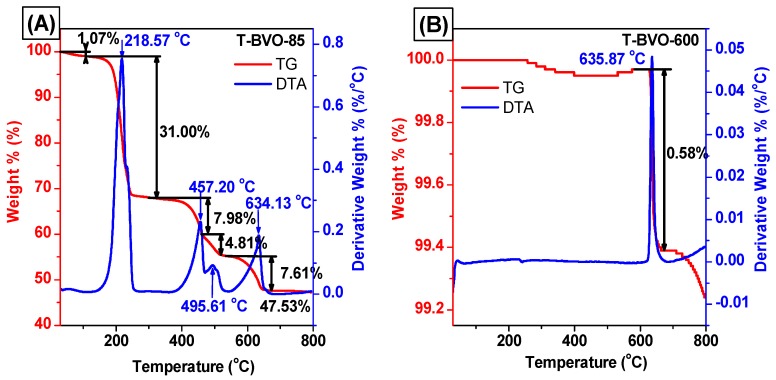
Differential thermal analysis-Thermal gravimetric analysis (DTA-TGA) curves of the BiVO_4_: (**A**) T-BVO-85 and (**B**) T-BVO-600.

TG analysis was performed to observe the physical and chemical processes that occur when the T-BVO-85 sample was processed at different temperatures as well as the purity of the T-BVO-600 sample. Figure 7 shows the weight loss curve of T-BVO-85 and T-BVO-600. From the TGA curve shown in Figure 7A, the T-BVO-85 has five mass loss processes occurring when the sample is heated from room temperature to 800 °C. The first loss of mass occurs from room temperature to 100 °C with a weight loss of 1.07%, corresponding to the removal of adsorbed water on the surface of the material. The second mass loss process in the range of temperature from 100 °C to 300 °C, related to the decomposition of thiourea, corresponds to approximately 31.00% of the weight loss. The third mass loss in the temperatures range from 300 °C to 470 °C is due to the decomposition of the nitrate salts with a mass loss of about 7.98% [47] The fourth mass loss in the temperature range from 470 °C to 540 °C involves complete oxidation of carbon residue in the sample with mass loss of about 4.81%. The fifth mass loss in the region from 540 °C to 680 °C is the transition between the tetragonal and monoclinic phases, which is accompanied by a weight loss of about 7.61%. The differential thermal analysis (DTA) curve of the T-BVO-85 shows five exothermic peaks, corresponding to five mass loss processes on the TG curve. The sharply exothermic peak at 218.67 °C with significant weight loss in the TG curve is attributed to the decomposition of the thiourea because the decomposition reaction of thiourea is the exothermic reaction. For the T-BVO-600 sample (Figure 7B), only one mass loss occurred during the high-temperature range of 605 °C to 690 °C, corresponding to the phase transition between the tetragonal and monoclinic phases. The weight loss accounts for about 0.68%. In addition, no other mass loss occurred at lower temperatures, indicating that thiourea and nitrate salts were decomposed entirely in the sample when the sample was heated at 600 °C.

### 3.3. Photocatalytic Activities

**Figure 8:**
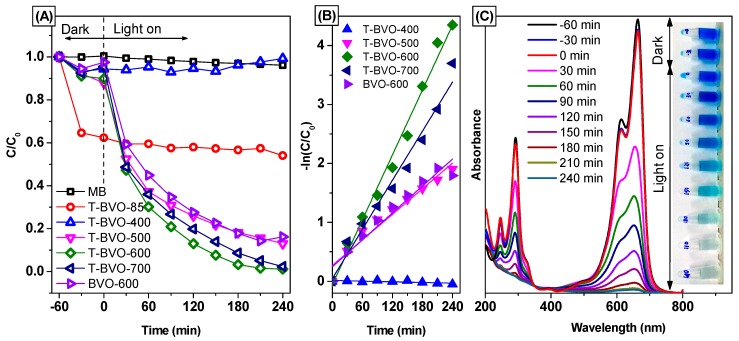
Photocatalytic degradation of methylene blue (MB) over BiVO_4_ samples (**A**), plots of ln(C_o_/C) versus irradiation time representing the fit using a pseudo-first-order reaction rate (**B**), and UV-vis absorption spectra of MB solution separated from catalyst suspensions during illumination using T-BVO-600, (**C**). Insert displays a digital photo of photodegradation for MB after different illumination times.

According to the results of the photocatalytic activity shown in Figure 8, it can be seen that on the synthetic BiVO_4_ samples using thiourea, the calcined samples showed better photocatalytic efficiency than the non-calcined BiVO_4_ sample (except for the sample calcined at 400 °C). After 240 minutes of irradiation, about 46.02% of MB was removed for the BiVO_4_ sample without calcining. However, the removal efficiency of MB can reach 87.13%, 98.93%, and 97.57% for the sample calcined at 500, 600, and 700 °C, respectively. The results show that calcining can enhance the photocatalytic activity of the thiourea-based BiVO_4_ sample, and the calcined sample at 600 °C has the best photocatalytic activity. For the non-thiourea synthesized BiVO_4_ calcined at 600 °C, low photocatalytic activity, only about 85.54% of MB was removed.

The photocatalytic degradation of MB according to the first kinetics [48], as confirmed by the linearity of ln(C_0_/C_t_) according to time (t, min) (shown in Figure 8B) and the reaction rate constants of the samples, are listed in Table 3. The results indicate that MB photolysis occurs very slowly with no catalyst. When using BiVO_4_ catalysts synthesized using thiourea, the photocatalytic activity of the samples increased as the calcining temperature increased, and the photocatalytic activity reaches a maximum when the calcining temperature is 600 °C (T-BVO- 600). The photocatalytic activity on the sulfate-modified BiVO_4_ increases in the following order: T-BVO-400, T-BVO-500, T-BVO-700, T-BVO-600 with the rate constant (k) respectively, are 1.881 × 10^−7^ min^−1^, 7.240 × 10^−3^ min^−1^, 13.90 × 10^−3^ min^−1^, and 18.37 × 10^−3^ min^−1^. The rate constant of BVO-600 is 7.620 × 10^−3^ min^−1^, smaller than that of the T-BVO-600 by about 0.548 times.

Figure 8C shows the change in the UV-vis absorption spectrum of MB over time in the presence of T-BVO-600. As the lighting time increases, the maximum absorption peak of MB at 664 nm decreases. The decrease in MB concentration was also observed through the dark blue of the MB solution, which began to fade, and the blue color was almost completely lost when the lighting time increased to 240 min. In addition, there is no increase in the absorption peak in the UV region of MB during irradiation, suggesting that most MB has completely decomposed. The high photocatalytic activity of T-BVO-600 indicates that it can be widely used in the treatment of wastewater containing organic dyes.

### 3.4. Investigation of the Mechanisms of Dye Degradation

**Figure 9:**
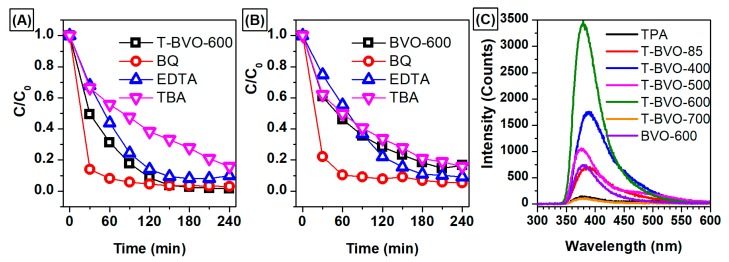
Trapping experiments of photocatalytic degradation of MB over (**A**) T-BVO-600, (**B**) BVO-600 samples (BQ: 1,4-benzoquinone, EDTA: ethylenediaminetetraacetic acid disodium and TBA: *tert*-butanol) and (**C**) PL spectra of terephthalic acid (λ_ex_ = 315 nm) in the presence of BiVO_4_ samples.

**Figure 10:**
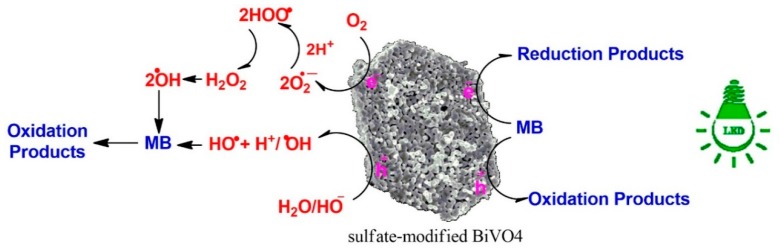
Illustrative representation of direct mechanism for MB photodegradation process.

The photodegradation mechanism of MB by T-BVO-600 and BVO-600 has been investigated via an indirect chemical probe method, using chemical agents to capture the active species produced during the early stages of photocatalysis. During photocatalytic oxidation, organic compounds (especially compounds containing double bonds) are attacked by active species, including holes (h^+^), hydroxyl radicals (HO^•^), and superoxide anion radical (O_2_^−^). According to previous studies, BQ, EDTA, and TBA were agents that capture O_2_^−^, h^+^, and HO^•^, respectively [49,50,51]. As shown in Figure 9A, the MB degradation effect of T-BVO-600 was only slightly reduced by the addition of EDTA and the decomposition efficiency decreased as TBA was added to the photocatalytic system. Meanwhile, the photocatalytic activity of MB degradation did not change significantly when EDTA and TBA were added to the reaction system (Figure 9B). These results indicate that h^+^ and HO^•^ are the major species of MB decomposition under T-BVO-600/visible light system. However, there was a slight increase in the MB degradation effect observed after BQ was added to the T-BVO-600/visible light system. This was also observed in the BVO-600/visible light system. This result is due to the increasing separation efficiency of electron-hole pairs through immediately e^−^ captured by BQ. The mechanism of the photocatalytic activity under visible light irradiation in the samples used in this study is described in Figure 10. The reaction mechanism can be proposed as follows:

Sulfate-modified BiVO_4_ + hν (visible light) → Sulfate-modified BiVO_4_ (e^−^(CB) + h^+^(VB))

H_2_O(ads) + h^+^(VB) → OH^•^(ads) + H^+^(ads)

O_2_ + e^−^(CB) → O_2_^−^(ads)

O_2_^−^(ads) + H^+^ → HOO^•^(ads) (2)

2HOO (ads) → H_2_O_2_(ads) + O_2_

H_2_O_2_ (ads) → 2OH^−^(ads)

MB + OH^−^ → dye intermediates → CO_2_ + H_2_O

MB + h^+^(VB) → dye intermediates → CO_2_ + H_2_O

In addition, the formation of hydroxyl radicals (HO^•^) on the surface of the BiVO_4_ catalyst was detected by photoluminescence (PL) technique using terephthalic acid as the sensor molecule. Terephthalic acid immediately reacts with the HO^•^ radicals to form 2-hydroxyterephthalic acid (HTA) with strong photoluminescence. Figure 9C shows the change in peak intensity of the reaction solution after 240 minutes of irradiation with the presence of BiVO_4_ samples under different synthesis conditions. In Figure 9C, the fluorescence signal is recorded with very low intensity in the wavelength range from 370 to 600 nm when no catalyst is used. However, in the presence of catalysts, strong fluorescence intensity was observed at 380 nm. The PL signal of the T-BVO-600 sample was higher than those of the other samples, indicating that the formation of HO^•^ on this sample was highest and correlated with the high photodegradation of MB.

### 3.5. Reusability and Stability

**Figure 11:**
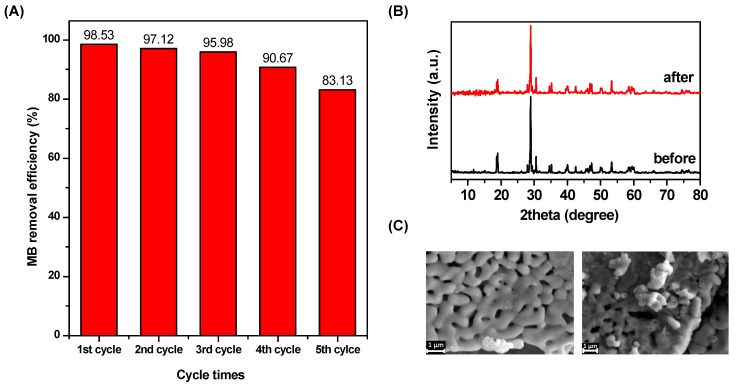
Photo-stability tests over T-BVO-600 sample for the cycling photodegradation of MB (**A**), XRD patterns (**B**), and SEM micrograph (**C**) of T-BVO-600 sample before and after the photo-stability tests.

To be an effective catalyst in practical applications, the reusability of the catalyst is a critical factor. Here, the reusability of the T-BVO-600 was tested five times. At each time, the reaction solution was withdrawn over time. The catalyst was separated by centrifugation and then collected and purified by washing (three times with ethanol and one time with distilled water) for the next experiment. The results are shown in Figure 11A. It can be seen that the photocatalytic activity of the material decreased gradually as the cycle was repeated. The removal efficiencies of MB at each cycle were 98.65%, 97.31%, 95.98%, 90.77%, and 83.11%, respectively. This result is due to the reduction of the catalyst in the purification process since micro-sized T-BVO-600 plates can adhere to the centrifuge tube causing sample loss during washings. Compared with the first cycle, the removal efficiencies of MB decreased slightly at the second and third times, and significantly decreased at the fourth and fifth times. In addition, the crystalline structure of the materials was also tested by XRD (Figure 11B). The XRD pattern of T-BVO-600 after five times of use still exhibits characteristic diffraction peaks as in monoclinic T-BVO-600 at a 2θ angle by 18.5°, 28.9°, 35°, and 47°. Also, the crystalline surface morphology before and after the reaction of T-BVO-600 is shown in Figure 11C. According to the SEM image, there is no clear difference in surface morphology and crystal structure. The results of the above analysis show that the crystal structure, as well as the morphology of the material, does not change after the photocatalytic reaction.

## 4. Conclusions

The sulfate-modified BiVO_4_ photocatalytic material with the high photocatalytic degradation efficiency of MB was successfully synthesized by a sol-gel method. The results indicate that the heat treatment exerted an important influence on the crystal phase, morphology, and crystallinity of BiVO_4_ when the BiVO_4_ was synthesized in the presence of thiourea. The thiourea also significantly affected the control of crystal formation and crystal phase of BiVO_4_ with and without the presence of thiourea and calcined at 600 °C. The as-prepared T-BVO-600 exhibited the highest degradation of MB, in which 98.53% removal of MB was achieved within 240 min. The T-BVO-600 exhibited good recyclability for MB removal, removal of MB was above 83% after five cycles. The T-BVO-600 with the features of high efficiency and good recycling ability is a promising photocatalyst for water purification.

## Figures and Tables

**Table 1:** Lattice parameters and the V–O bond lengths at 829 cm^−1^ for the Bismuth vanadate (BiVO_4_) prepared by the different annealing temperature.

Sample No.	Preparation Condition (°C)	Cell Parameters ^a^					Crystallite Size ^a^ (nm)	Bond Length ^b^ (Å)
		a	b	c	β	V_cell_		
		(Å)	(Å)	(Å)	(Å)	(Å^3^)		V–O
T-BVO-85	85	−	−	−	−	−	−	−
T-BVO-400	400	5.136	5.094	11.686	90.257	305.277	36.890	1.7002 ± 0.0005
T-BVO-500	500	5.174	5.101	11.664	90.222	307.809	34.420	1.6961 ± 0.0002
T-BVO-600	600	5.186	5.093	11.669	90.174	308.195	33.700	1.6940 ± 0.0002
T-BVO-700	700	5.192	5.094	11.667	90.198	308.576	32.740	1.6937 ± 0.0006
BVO-600	600 (without thiourea)	5.192	5.095	11.664	90.206	308.501	33.190	1.6935 ± 0.0006

^a^ Data obtained by XRD data; ^b^ Data obtained by Raman data.

**Table 2:** Surface element compositions of the BVO-600 and T-BVO-600 samples.

Sample No.	Atomic % ^a^				
	O 1s	C 1s	Bi 4f	V 2p3	S 2s
BVO-600	46.55	29.25	15.85	8.36	−
T-BVO-600	50.94	28.44	11.67	6.22	2.72

^a^ Data obtained by XPS data

**Table 3:** The bandgap energy of the samples and the rate constants (k) values of the samples for MB degradation.

Sample No.	Preparation Condition (°C)	Bandgap ^c^ (E_g_)	First Kinetics			
			K × 10^−3^	R^2^	t_1/2_	t_90_
		eV	(min^−1^)		(min)	(min)
T-BVO-85	85	−	4.313 × 10^−4^	0.824	1.607 × 10^6^	5.339 × 10^6^
T-BVO-400	400	2.230	1.881 × 10^−4^	0.506	3.685 × 10^6^	12.241 × 10^6^
T-BVO-500	500	2.112	7.240	0.955	95.738	318.037
T-BVO-600	600	2.288	18.370	0.989	37.733	125.345
T-BVO-700	700	2.277	13.900	0.977	49.867	165.654
BVO-600	600 (without thiourea)	2.246	7.620	0.945	90.964	302.176

^c^ Data obtained by UV-Vis-DRS data; ^d^ Data obtained by the relationship between ln (C_0_/C) and irradiation time t (min)
